# Relatives of Sick or Wounded Soldiers to Be Advised of Latter’s Condition

**Published:** 1918-09

**Authors:** 


### Relatives of Sick or Wounded Soldiers to Be Advised of Latter's Condition.

---

Washington, August 16.—Exact information concerning wounded and sick American soldiers admitted to hospitals overseas will be made immediately available to relatives or friends of the men, under a plan being worked out at the war department.

Secretary Baker said today he had visited the office of Surgeon General Gorgas to look into the daily reports from the hospitals, with a view to having them carded, catalogued and tabulated, so that the most instant information can be given to all inquirers.

The hospital records, Mr. Baker said, will be brought here weekly by courier from France, and thus it will be possible to give the exact nature of the wound or the disease promptly from which the men are suffering. The information will be available through the adjutant general.

The task of installing the system will be a big one, but the war secretary believes the information should be available, for in thousands of cases it will relieve unnecessary distress and doubt which follow appearance of the names of men on casualty lists as wounded, degree undetermined, or severely.

